# Establishment of age- and gender-specific pediatric reference intervals for liver function tests in healthy Han children

**DOI:** 10.1007/s12519-018-0126-x

**Published:** 2018-03-15

**Authors:** Xin Li, Di Wang, Chun Yang, Qi Zhou, Suo-Lang Zhuoga, Li-Qiang Wang, Han-Xin Yao, Qin Zhang, Qing Ai, Chen-Xi Yang, Jian-Cheng Xu

**Affiliations:** 1 Department of Laboratory Medicine, First Hospital of Jilin University, Xinmin Street No. 71, Changchun, 130021 China; 2 Department of Pediatrics, First Hospital of Jilin University, Changchun, China; 3 Centre for Heart & Lung Innovation, University of British Columbia, Vancouver, BC Canada

**Keywords:** Age, Gender, Liver function tests, Pediatric, Reference intervals

## Abstract

**Background:**

The development and growth of children influence values of liver function tests. This study aims to establish age- and gender-specific pediatric reference intervals of liver function among Han children in Changchun, China.

**Methods:**

A total of 1394 healthy Han children, aged 2–14 years, were recruited from communities and schools with informed parental consent in Changchun. The levels of serum alanine aminotransferase (ALT), aspartate aminotransferase (AST), γ-glutamyltransferase (GGT), alkaline phosphatase (ALP), total protein (TP), albumin (ALB), total bilirubin (TBIL) and direct bilirubin (DBIL) were measured on Hitachi 7600-210 automatic biochemical analyzer. The age- and gender-specific reference intervals were partitioned using Harris and Boyd’s test and calculated using nonparametric rank method. The pediatric reference intervals were validated in five representative hospitals located in different areas in Changchun.

**Results:**

All the analytes required some levels of age partitioning. Proteins (TP, ALB) and bilirubins (TBIL, DBIL) required no gender partitioning. In contrast, considerable gender partitioning was required for serum ALT, AST, GGT, and ALP. TP, TBIL, and DBIL showed steady increases, and AST showed apparent decreases over time, whereas ALT, GGT, ALP, and ALB demonstrated complex trends of change. ALT and GGT increased sharply in males from 11 to 14 years old. However, ALP declined in females from 13 to 14 years. All five laboratories passed the validation of reference intervals.

**Conclusions:**

There were apparent age or gender variations of the reference intervals for liver function. When establishing pediatric reference intervals, partitioning according to age and gender is necessary.

## Introduction

Liver function tests, including assays for alanine aminotransferase (ALT), aspartate aminotransferase (AST), alkaline phosphatase (ALP), γ-glutamyltansferase (GGT), total protein (TP), albumin (ALB), total bilirubin (TBIL), and direct bilirubin (DBIL), are generally ordered as a “test of exclusion” in patients with non-specific symptoms or as part of routine health checks. In children, reference intervals of liver function tests should reflect the different phases of physiological development from birth to adolescence. However, appropriate age- and gender-specific pediatric reference intervals for most tests are often incomplete.

Reference intervals are basic dimension to explain laboratory tests and analyze tests information. Proper and accurate laboratory tests and reference intervals are vitally necessary for medical assessment and care of patients. Current guidelines define a reference interval as the interval between two values in which 95% of the results for apparently healthy individuals would fall, usually between the 2.5 and 97.5 centiles of the distribution of test results for a reference population [1].

For several years, there are few systematic reference interval studies based on Chinese people. The reference intervals now applied in Chinese laboratory are mainly based on European and American populations study tens of years ago. Until now, there are still no reference intervals about pediatric population. As a completely different group, children have different physical size, organ maturity, nutrition and metabolism status with adults. Thus, it is inappropriate for children and adult to use a common reference interval. Pediatric reference interval should reflect children’s different phases of physiological development from birth to adolescence which can profoundly influence the concentrations of liver function tests routinely measured in the clinical laboratory.

Recent studies including the Canadian Laboratory Initiative on Pediatric Reference Intervals (CALIPER) and reports from America, Sweden and Taiwan of China have added some novel information [2–5]. For example, the CALIPER has established a comprehensive covariate-stratified reference intervals based on a healthy nonhospitalized, and multiethnic pediatric population [3]. However, it is not appropriate to directly apply these reference intervals to Chinese pediatric population, because some parameters may vary significantly among different races and regions. Furthermore, several studies have decided to recruit participants who have been hospitalized or approximately “healthy” individuals due to the difficulties recruiting study participants [6–8]. Nevertheless, with a view to physical, nutritional, and developmental status, it is also not appropriate to apply these reference intervals to common pediatric population. Thus, taking into account finding from these studies, the present study will recruit children from communities and schools for conventional check-up before starting entering nursery, kindergarten, elementary school, and middle school. Moreover, taking into account other significant covariates of age, gender, region and ethnicity on pediatric reference intervals, this study presents age- and gender-specific pediatric reference intervals for liver function tests based on definitely healthy reference individuals in Changchun, China.

## Methods

### Subjects

From February 2015 to March 2016, healthy children aged from 2 to 14 years were recruited in this study. In order to obtain samples from healthy children, the recruitment of study population aged from 2 to 5 years took place in the community and health-care center (Lvyuan Maternal and Children Health Hospital) for common physical examination which national rules require before going to nursery. The children with age range of 6–14 years were enrolled in primary school (Lvyuan District Primary School of Changchun) and middle school (No. 78 Middle High School of Changchun). All the serum samples were collected after obtaining parental permission. All the potential participants or their parents completed a questionnaire including questions on diseases, prescribed and over-the-counter medication, the presence of fever, allergy and eczema, and a general question concerning subjective health. The demographic data included diet, exercise status, ethnicity, and body mass index parameters. Participants were excluded if they had a history of illness such as acute infection, metabolic or any system disease, surgery experiences within 6 months, use of prescription medications over the previous 2 weeks. The included children would be further removed according to the following testing criterion: positive in hepatitis B surface antigen, hepatitis c virus antibody, and human immunodeficiency virus antibody; creatinine above 120 μmol/L; creatine kinase above 500 U/L; uric acid above 475 μmol/L; glucose above 7.0 mmol/L; C reaction protein above 12.0 mg/L. Therefore, a total of 1394 individuals, 649 females and 745 males, were included.

This study was approved by the institutional ethics committee of the First Hospital of Jilin University. For all the children, their parents provided written informed consent and when possible, child subjects provided written assent.

### Specimen collection and handling

All participants kept a normal diet and exercise in the last 3 days. Before blood collection in the morning, each participant fasted for at least 8 hours over night. Samples were collected in serum separator tubes (SST™; BD), then centrifuged, separated and aliquoted within 2 hours of collection. Excluding unqualified samples which were hemolysis, lipidemia, and jaundice, all qualified samples were sent to the laboratory for examination as soon as possible.

### Instruments, reagents and methods

All 8 liver function tests were performed on Hitachi 7600-210 automatic analyzer (Hitachi High-Technologies, Tokyo, Japan). ALT (UV-LDH method), AST (UV-MDH method), GGT (l-γ-glutamyl-3-carboxy-4-nitranilide method), and ALP (AMP buffer method) were measured using reagents from Kehua bio-engineering company (Shanghai, China); TP (biuret reaction), ALB (bromocresol green method), TBIL, and DBIL (DCA method) were measured using reagents from DiaSys Diagnostics (Shanghai, China).

### Quality control

Sample determinations were carried out in the Department of Laboratory Medicine at the First Hospital of Jilin University. The Laboratory was accredited to ISO 15189:2012 Medical Laboratories-Particular Requirements for Quality and Competence by China National Accreditation Service for Conformity Assessment in 2012. All the doctors, technicians, and nurses participating in the physical examination and sample analysis were through strict training. Hitachi 7600-210 automatic analyzer were calibrated per 12 months by the manufactures, so as to ensure the performance of detection system like precision, accuracy, analytical measurement range, clinical reportable range and carryover rate were controlled.

### Validation of reference intervals

The established pediatric reference intervals are based on the population living in Changchun. As recommended by the Clinical and Laboratory Standards Institute (CLSI) C28-A3 [1], the reference intervals need to be assessed applicability for different testing conditions. We performed the validation in five representative hospitals located in different areas in Changchun including Lab 1 (The First Hospital of Jilin University), Lab 2 (The branch, First Hospital of Jilin University), Lab 3 (The Second Hospital of Jilin University), Lab 4 (The Fourth Hospital of Jilin University), and Lab 5 (The Pediatric Hospital of Changchun). For each subgroup of reference intervals 20 healthy participants were recruited regionally for each of the five hospitals. Routine instruments, reagents and methods were used to test samples from recruited individuals in each laboratory. For each reference subgroup, the results of analytes were validated with the newly established reference intervals. If no more than 10% of the results outside the limits, the established reference interval was considered applicable for the population.

### Statistics analysis and determination of reference intervals

Statistical analysis was performed with EXCEL (Microsoft) and SPSS 21.0 (IBM) software and in accordance with CLSI C28-A3 guideline [1]. Briefly, the data were inspected using scatter and distribution plots; outliers were removed with the Dixon’s rule; distribution was identified with the Shapiro–Wilk Statistic. Age and gender partitions were first determined by visually inspecting the distribution and scatter plots for overall trends. It was then justified with Harris and Boyd’s test which is recommended currently by the CLSI [1]. According to Harris and Boyd’s method [9], we calculated *Z* values with the standard deviation and a modified *Z*-statistic for two groups to determine whether each group is sufficiently different statistically to its own grouping. If the results of Harris and Boyd’s test did not demonstrate partition, the two groups were then combined and revaluated. The reference interval of each group was calculated using the nonparametric method to calculate the lower and higher reference limits, respectively. For each reference interval, 90% confidence intervals were calculated for either ends.

## Results

A total of 1394 samples from 649 males and 745 females (2–14 years) were used to calculate age- and gender-specific reference intervals, and the male: female ratio was 1:1.15. The 8 liver function tests in this study were measured on Hitachi 7600-210 automatic biochemical analyzer, along with the assay of detection, as specified in the manufacturer’s package insert. The cumulative frequency of each measured value for each partition was calculated. After removing outliers, the value for each partition followed Gaussian distribution. With the Harris and Boyd’s test, we observed that it was necessary to derive separate reference intervals by age and gender in children. For each reference interval, 90% confidence intervals were calculated for either ends. Table 1 shows the 2.5th and 97.5th percentiles for 8 liver function tests (serum enzymes, proteins, and bilirubins) of each age and gender group. Table 2 includes both the calculated age- and gender-specific reference intervals for respective analytes of combined groups and points for each interval. As Table 1 shows, most analytes required some amount of partitionings, by age, gender, or both. There were apparent age variations of the reference intervals for all 8 liver function tests. Interestingly, all four enzymes required no gender partition within the initial several age groups but required in the later age groups. However, bilirubins (TBIL, DBIL) and proteins (TP, ALB) displayed no gender partition in the whole groups. All the analytes required a minimum of 2 age-specific reference intervals. ALB only required two different age partitions: 2–10 years, and 11–14 years. However, AST required 7 different age- and gender-specific partitions: 2 years, 3–5 years, 6 years, 7–12 years (female), 7–12 years (male), 13–14 years (female), and 13–14 years (male).

**Table 1:** Age and gender specific 2.5th and 97.5th percentile results for 8 liver function tests in healthy children aged 2–14 years (*n *= 1394)

Age (y)	Sex	*n*	ALT (U/L)		AST (U/L)		GGT (U/L)		ALP (U/L)		TP (g/L)		ALB (g/L)		TBIL (µmol/L)		DBIL (µmol/L)	
			2.5	97.5	2.5	97.5	2.5	97.5	2.5	97.5	2.5	97.5	2.5	97.5	2.5	97.5	2.5	97.5
2	M	109	8	22	27	49	8	15	174	361	59.5	73.2	40.1	53.3	2.7	9.3	0.9	2.6
	F	87	9	25	27	47	7	16	173	426	61.6	72.0	41.4	51.9	3.3	14.0	0.9	3.6
3	M	135	7	25	26	45	7	17	156	364	60.8	74.6	41.1	52.5	3.0	13.8	1.0	3.5
	F	121	9	22	20	42	8	16	162	365	59.2	73.7	39.7	53.1	3.2	10.6	0.8	2.9
4	M	79	7	20	25	39	8	15	148	337	60.3	76.3	40.8	50.5	3.9	15.2	0.9	3.8
	F	72	7	19	20	44	8	16	150	326	53.7	75.9	39.7	51.2	3.2	16.3	1.1	4.0
5	M	78	7	22	25	44	9	20	144	369	60.9	76.9	40.3	52.5	4.4	13.6	1.4	3.7
	F	75	6	21	21	41	7	19	143	360	59.8	74.0	39.7	52.2	3.9	12.4	1.3	3.2
6	M	57	6	23	20	40	9	17	152	356	58.8	75.2	40.7	52.1	4.1	11.8	1.2	3.7
	F	46	6	23	19	39	8	16	180	372	59.9	76.8	38.6	50.1	3.4	12.9	0.9	3.2
7	M	43	4	18	8	35	10	16	129	359	63.9	77.1	41.2	51.4	2.8	13.5	1.1	3.6
	F	35	4	16	10	37	8	16	136	345	63.8	78.5	40.9	53.3	3.6	14.9	1.2	3.6
8	M	32	4	18	16	34	9	21	153	333	61.1	76.2	40.8	50.6	5.4	14.1	1.9	4.0
	F	23	6	20	14	30	7	22	165	290	66.4	73.2	42.8	47.6	5.5	12.5	1.6	3.7
9	M	24	6	18	18	30	7	19	144	367	65.4	74.6	41.7	51.9	6.0	14.6	1.8	4.4
	F	24	5	16	14	34	5	25	151	351	64.7	75.0	42.3	49.0	6.9	12.6	1.9	3.6
10	M	41	4	19	16	30	8	20	151	359	62.2	76.7	41.0	50.2	5.2	15.3	1.7	4.5
	F	53	3	19	13	29	7	22	137	462	59.4	79.1	39.0	50.2	4.0	16.6	1.6	5.3
11	M	29	5	33	14	35	11	27	153	412	55.8	81.3	37.4	52.8	5.3	15.4	1.7	4.3
	F	27	6	17	12	34	10	27	110	366	62.5	77.5	39.9	51.6	4.6	13.6	1.6	4.3
12	M	33	5	30	15	42	10	45	160	516	65.4	79.0	41.0	53.0	5.7	12.6	1.8	3.5
	F	30	5	18	13	29	9	22	97	354	65.7	78.3	42.2	51.8	4.5	15.0	1.6	4.1
13	M	31	7	33	15	36	10	32	134	437	64.0	77.2	43.4	51.8	5.1	15.4	1.5	3.8
	F	23	5	16	15	22	8	18	68	224	66.2	76.4	43.2	51.2	3.4	21.3	0.9	5.3
14	M	54	7	35	14	30	10	40	126	424	64.7	81.9	41.3	53.7	4.5	16.0	1.7	4.3
	F	33	5	30	14	23	9	33	49	152	67.6	78.2	42.7	51.9	4.4	18.4	2.0	5.3

*ALT* alanine aminotransferase,* AST* aspartate aminotransferase,* GGT* γ-glutamyltransferase,* ALP* alkaline phosphatase,* TP* total protein,* ALB* albumin, *TBIL* total bilirubin,* DBIL* direct bilirubin,* M* male,* F* female

**Table 2:** Age- and gender-specific reference intervals for 8 liver function tests in healthy children aged 2–14 years (*n *= 1394)

Analytes	Age group (y)	Sex group	No. of samples	Lower limit	Upper limit	Confidence interval for lower limit	Confidence interval for upper limit
ALT (U/L)	2	F + M	196	8	24	7–9	22–26
	3 to < 6	F + M	663	7	23	6–7	21–24
	7 to < 10	F + M	275	4	19	3–4	16–22
	11 to < 14	F	113	5	25	4–6	15–33
		M	147	5	35	4–6	31–37
AST (U/L)	2	F + M	196	27	49	25–29	45–53
	3 to < 5	F + M	560	22	44	20–24	42–45
	6	F + M	103	18	40	16–21	38–42
	7 to < 12	F	192	11	35	10–12	31–38
		M	202	13	39	10–16	33–42
	13 to < 14	F	59	14	26	13–15	18–28
		M	82	13	36	13–15	26–40
GGT (U/L)	2 to < 4	F + M	603	8	16	7–8	15–17
	5 to < 10	F + M	530	8	21	7–9	19–22
	11 to < 14	F	113	8	29	7–9	17–41
		M	147	10	43	9–11	35–51
ALP (U/L)	2 to < 3	F + M	452	160	376	153–169	355–390
	4 to < 9	F + M	587	143	362	134–153	354–370
	10 to < 12	F + M	213	116	426	99–136	354–471
	13 to < 14	F	56	48	232	42–58	149–243
		M	85	111	486	92–148	338–512
TP (g/L)	2 to < 3	F + M	452	59.5	74.4	59.1–60.1	72.9–75.3
	4 to < 6	F + M	407	59.2	76.4	57.2–61.2	75.6–77.3
	7 to < 10	F + M	275	62.5	78.2	59.1–64.1	76.2–80.2
	11 to < 14	F + M	260	63.6	79.2	61.5–66.1	77.4–80.8
ALB (g/L)	2 to < 10	F + M	1134	40.0	52.3	39.7–40.5	51.9–52.7
	11 to < 14	F + M	260	41.3	53.1	39.3–43.5	52.5–53.5
TBIL (µmol/L)	2 to < 3	F + M	451	3.0	13.0	2.8–3.2	11.9–13.7
	4 to < 7	F + M	481	3.4	14.8	3.2–3.8	13.9–15.7
	8 to < 14	F + M	455	4.5	16.6	4.0–5.0	15.6–17.6
DBIL (µmol/L)	2 to < 3	F + M	451	0.9	3.3	0.8–1.0	3.1–3.5
	4 to < 7	F + M	481	1.1	3.8	1.0–1.2	3.5–3.9
	8 to < 12	F + M	316	1.6	4.5	1.5–1.8	4.1–4.9
	13 to < 14	F + M	139	1.2	5.3	0.8–1.6	4.8–5.9

*ALT* alanine aminotransferase,* AST* aspartate aminotransferase,* GGT* γ-glutamyltransferase,* ALP* alkaline phosphatase,* TP* total protein,* ALB* albumin, *TBIL* total bilirubin,* DBIL* direct bilirubin,* M* male,* F* female

**Fig. 1:**
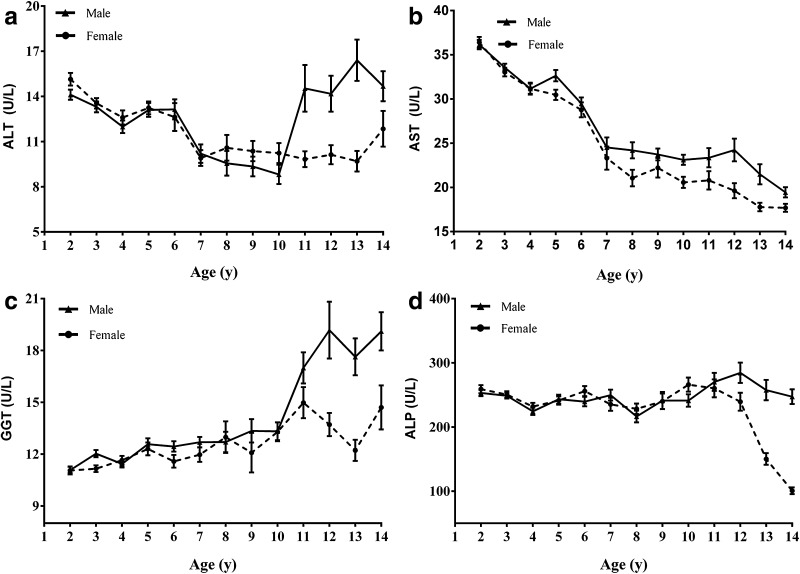
Trends in analyte concentrations of serum ALT, AST, GGT, and ALP in healthy males and females aged 2–14 years (*n *= 1394). Levels of serum ALT (**a**), AST (**b**), GGT (**c**), and ALP (**d**) in healthy children. Data are presented as mean ± SE. *ALT* alanine aminotransferase, *AST* aspartate aminotransferase, *GGT* γ-glutamyltransferase, *ALP* alkaline phosphatase, *SE* standard error

**Fig. 2:**
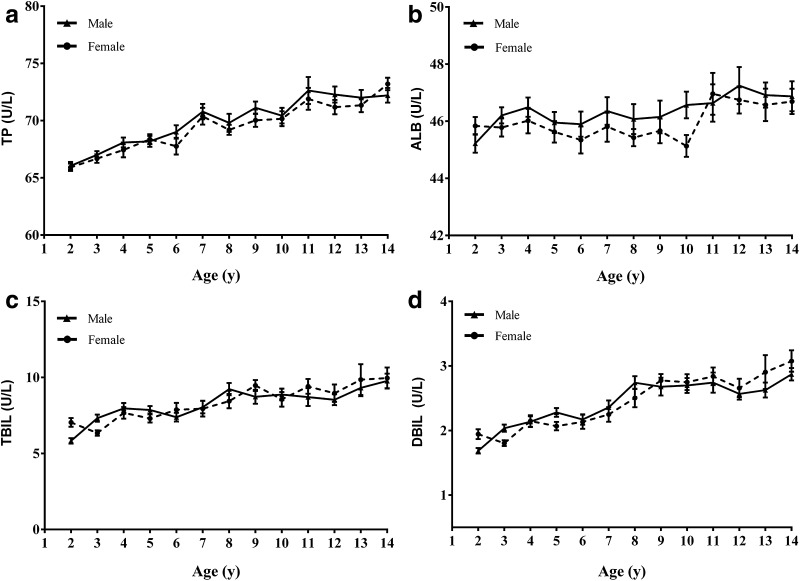
Trends in analyte concentrations of serum TP, ALB, TBIL, and DBIL in healthy males and females aged 2–14 years (*n *= 1394). Levels of serum TP (**a**), ALB (**b**), TBIL (**c**), and DBIL (**d**) in healthy children. Data are presented as mean ± SE. *TP* total protein, *ALB* albumin, *TBIL* total bilirubin, *DBIL* direct bilirubin, *SE* standard error

Within these analytes, TP, TBIL, and DBIL showed steady increases, and AST showed apparent decreases in analytes concentration over time, whereas ALT, GGT, ALP, and ALB demonstrated complex trends of change in these time (Figs. 1, 2). ALT and GGT increased sharply in males from 11 to 14 years. However, ALP markedly declined in females from 13 to 14 years.

As Table 3 shows, all five laboratories passed the validation for each age and gender subgroup of pediatric reference intervals and all the validation data fell within the range of intervals for those 8 liver function analytes. The validation results indicate that the newly reference intervals are applicable in most representative laboratories in Changchun.

**Table 3:** The results of reference interval validation in 5 laboratories

Analytes	Age group (y)	Sex group	Reference intervals	*N* ^a^	Lab 1		Lab 2		Lab 3		Lab 4		Lab 5	
					*n* ^b^	Result, %^c^	*n* ^b^	Result, %^c^	*n* ^b^	Result, %^c^	*n* ^b^	Result, %^c^	*n* ^b^	Result, %^c^
ALT (U/L)	2	F + M	8–24	20	2	90	1	95	1	95	1	95	1	95
	3 to < 6	F + M	7–23	20	2	90	2	90	2	90	2	90	1	95
	7 to < 10	F + M	4–19	20	2	90	2	90	2	90	2	90	0	100
	11 to < 14	F	5–25	20	1	95	0	100	2	90	2	90	0	100
		M	5–35	20	1	95	0	100	0	100	0	100	1	95
AST (U/L)	2	F + M	27–49	20	2	90	2	90	1	95	2	90	2	90
	3 to < 5	F + M	22–44	20	0	100	2	90	0	100	2	90	1	95
	6	F + M	18–40	20	0	100	1	95	0	100	0	100	0	100
	7 to < 12	F	11–35	20	1	95	2	90	0	100	1	95	1	95
		M	13–39	20	0	100	2	90	0	100	2	90	0	100
	13 to < 14	F	14–26	20	1	95	2	90	2	90	2	90	0	100
		M	13–36	20	1	95	1	95	0	100	2	90	1	95
GGT (U/L)	2 to < 4	F + M	8–16	20	0	100	0	100	0	100	2	90	2	90
	5 to < 10	F + M	8–21	20	1	95	0	100	0	100	2	90	1	95
	11 to < 14	F	8–29	20	0	100	2	90	2	90	2	90	0	100
		M	10–43	20	1	95	0	100	2	90	0	100	2	90
ALP (U/L)	2 to < 3	F + M	160–376	20	2	90	2	90	2	90	2	90	0	100
	4 to < 9	F + M	143–362	20	1	95	2	90	0	100	1	95	0	100
	10 to < 12	F + M	116–426	20	0	100	2	90	0	100	2	90	2	90
	13 to < 14	F	48–232	20	1	95	1	95	2	90	2	90	2	90
		M	111–486	20	0	100	2	90	0	100	2	90	2	90
TP (g/L)	2 to < 3	F + M	59.5–74.4	20	1	95	2	90	0	95	2	90	1	95
	4 to < 6	F + M	59.2–76.4	20	0	100	1	95	0	100	2	90	0	100
	7 to < 10	F + M	62.5–78.2	20	0	100	2	90	0	100	2	90	1	95
	11 to < 14	F + M	63.6–79.2	20	0	100	1	95	0	100	2	90	2	90
ALB (g/L)	2 to < 10	F + M	40.0–52.3	20	0	100	2	90	0	100	2	90	0	100
	11 to < 14	F + M	41.3–53.1	20	0	100	2	90	0	100	2	90	0	100
TBIL	2 to < 3	F + M	3.00–13.01	20	0	100	1	95	1	95	2	90	0	100
(µmol/L)	4 to < 7	F + M	3.41–14.80	20	0	100	1	95	0	100	1	95	0	100
	8 to < 14	F + M	4.50–16.60	20	0	100	2	90	0	100	2	90	1	95
DBIL	2 to < 3	F + M	0.90–3.30	20	0	100	1	95	2	90	2	90	0	100
(µmol/L)	4 to < 7	F + M	1.10–3.80	20	0	100	2	90	1	95	2	90	0	100
	8 to < 12	F + M	1.60–4.50	20	2	90	2	90	2	90	2	90	2	90
	13 to < 14	F + M	1.20–5.30	20	1	95	2	90	1	95	2	90	0	100

*ALT* alanine aminotransferase,* AST* aspartate aminotransferase,* GGT* γ-glutamyltransferase,* ALP* alkaline phosphatase,* TP* total protein,* ALB* albumin, *TBIL* total bilirubin,* DBIL* direct bilirubin,* M* male,* F* female. ^a^Number for validation samples of this study. ^b^Number for validation samples outside the reference intervals of this study. ^c^The results of percentage of validation samples inside the reference intervals of this study

## Discussion

Age- and gender-specific pediatric reference intervals were established non-parametrically for 8 liver function tests assayed on Hitachi 7600-210 automatic analyzer, sampled from 1394 healthy children in Changchun. This is a significantly larger reference set than used in other studies of this type [10–13].

Defining pediatric reference intervals is one of the most difficult tasks for physicians because of the difficulties in collection of blood samples from a sufficient large number of healthy children. Such difficulties are reflected by the fact that recent studies obtained samples by collecting hospital-based results or using surplus serum that come from children attending selected out-patient clinics, such as dentistry, plastic surgery, orthopedics [6–8]. CALIPER initiative has ever established pediatric reference intervals derived from the analysis of outpatient clinics but deemed to be metabolically stable [14]. However, we can not certain whether nor not these samples were free of affecting the measured analytes parameters. In the present study, we collected direct samples from children who were accepting common physical-examination in community health-care centers, schools and fitted into by questionnaires and the following inclusion criteria. In some periods of life such as neonates (0–1 month old) and the first year of life (1 year old), the continuously developing physiology of growing changes their laboratory values. Unfortunately, data from the short phase after birth was lacking and puberty aged from 13 to 14 years was insufficient in the present study.

In the present study, age-related changes in analyte concentrations were observed more commonly than gender associated differences. These analytes showed multiple separated age-related reference intervals. The verifying reference intervals established in the current study reflected pediatric development and growth changes during childhood. Within the analytes studied, proteins (TP, ALB) and bilirubins (TBIL, DBIL) reported no gender partitioning. Therefore, only age is required. This finding is consistent with previous studies for 2–14 years pediatrics subjects [3, 7, 15].

Except for age-related intervals, four enzymes, ALT along with AST, GGT and ALP, required additional gender-stratified reference intervals. ALT and AST do not reveal a distinct gender dependency during the childhood, for ALT in males an obvious increase up from 11 years to juveniles can be seen (Fig. 1). This finding differs from a previous study which described reference intervals for ALT according to gender throughout 2–14 years old but no age-related difference [7]. For AST, a continuous decrease in concentration in children from 2 to 14 years was also observed and showed gender differences (Fig. 2) which was consistent with previous studies [3, 16, 17]. ALP showed gender difference from 13 years old and was different from previous studies [8, 18]. ALP has also been known to reflect height velocity [19]. In this study, the average age at peak height velocity is 11–12 years in females. A marked decrease of ALP in females aged 13–14 years may reflect a reduced height velocity. However, previous studies reported different age class of gender difference [7, 8, 18]. The deviations from study to study and area to area highlight the importance of determining ethnicity specific intervals.

Although previous studies indicated that the levels of serum TP and ALB differed between males and females in certain age groups [3, 20], our data did not show significant statistic difference in gender groups. Furthermore, considerable age partitioning was also required for the proteins (TP, ALB) as well as bilirubins (TBIL, DBIL) and enzymes (ALT, AST, GGT, and ALP). For each of these analytes, they required a minimum of two age-specific reference intervals. Within these groups of analytes, TP, TBIL, DBIL, AST and GGT showed steady increases or decreases in concentration throughout time, whereas ALT, ALB, ALP showed a complex age-related pattern of change (Figs. 1, 2). This marked changes and fluctuations in children during their growth and development highlight the importance of establishing age-specific pediatric reference intervals.

All five laboratories passed the reference interval validation for each liver function analyte in this study. The results suggest that the newly established pediatric reference intervals are valid for most of the children in Changchun and can be used directly by most of the hospitals with guaranteed accuracy. However, considering the different populations and nationalities from other areas in Jilin province, we have already started to recruit reference individuals from more areas in Jilin province for further validation. We aim to establish pediatric reference intervals for major population who under treatment in Changchun.

Although the partitions of reference intervals are required based on firm statistical analysis, it is unclear whether these statistic differences are medical relevant. Thus, the reference interval data need to be updated every few years as the reference population change with environmental and nutrition factors. Besides, the diversity of detection system could lead to differences of test results. In the present study, these intervals are Hitachi 7600 method specific. They might need to be transferred when used in any laboratory that requires pediatric reference intervals if they are transferable to different methods as recommended by CLSI C28-A3 [1]. Completion of these transferences will allow a broader application of reference intervals developed through our study. The new database may also be of rural benefit and likely be used by hospital laboratories in other cities, although intervals should also be validated with local populations and transferred with recommendation by the CLSI [1].

In summary, this study provided updated age- and gender-specific pediatric reference intervals for 8 basic liver function tests using a modern analytical chemistry platform and a large cohort study of healthy children. All analytes required age partitioning. Proteins (TP, ALB) and bilirubins (TBIL, DBIL) did not require gender. In contrast, considerable gender partitioning was required for ALT, AST, GGT, and ALP. The marked changes and fluctuations in children during their growth and development make it important to determine age- and gender-pediatric reference intervals and, therefore, enhance the diagnostic safety in clinical laboratory.
